# Supplementary material for: Building clone-consistent ecosystem models
Source: PLoS Comput Biol. 2021 Feb 8;17(2):e1008635. doi: 10.1371/journal.pcbi.1008635 (PMC7895417; doi:10.1371/journal.pcbi.1008635)
Supplement: S1 Appendix — (PDF) [file pcbi.1008635.s001.pdf]

S1 Appendix: Further Analysis of UTI Models

Gerrit Ansmann and Tobias Bollenbach
Institute for Biological Physics, University of Cologne, Zülpicher Straße 77, 50937 Köln, Germany

A. Inconsistencies in the model from Ref. 1

We here provide explicit examples for an inconsistent behavior of the model described by Eq. 21:

$$\dot{x}_j = x_j \underbrace{g_j \left\lceil 1 + \sum_{i\neq j} a_{ji} x_i \right\rceil}_{\text{growth term}} \underbrace{\left(1 - \frac{x_j}{\left\lceil 1 + \sum_{i\neq j} b_{ji} x_i \right\rceil}\right)}_{\text{carrying-capacity term}}.$$

First, in Fig. S1 we show a simulation similar to Fig. 1 exhibiting different results when simulating the same scenario in two different ways. This simulation also exposes another problem of the model, namely that it contradicts the underlying assumption that a population also represents its footprint. Without dilution, this footprint cannot decrease and thus populations cannot decrease, which however happens in the simulation in Fig. S1.

Second, in the following we provide an arithmetic example that features no growth interaction and thus does not rely on how we choose identical populations to affect each other's growth (see Section B) – a choice that is not clear without experiment. We consider the case of three populations $\{1, 2, 3\} =: J$ with the first two populations having identical properties. We choose $g_j = 1$ for all $j \in J$, $a_{j,k} = 0$ for all $j, k \in J \times J$, i.e., the growth term is not affected by interaction and is always 1. Finally, we let the coefficients of the capacity term be:

$$b = \begin{pmatrix} \cdot & -1 & \frac{1}{4} \\ -1 & \cdot & \frac{1}{4} \\ \frac{1}{4} & \frac{1}{4} & \cdot \end{pmatrix}.$$

$b_{12} = -1$ and $b_{21} = -1$ reflects that two populations with identical properties fully occupy each other's niches. Now, consider two states of the community $x = \left(\frac{1}{4}, \frac{1}{4}, 1\right)$ and $\hat{x} = \left(\frac{1}{2}, 0, 1\right)$. As the first two populations are indistinguishable, these states describe an equivalent situation. Thus, they should also evolve equivalently, i.e., the temporal derivative of the summed populations 1 and 2 should be the same in both cases: $\dot{x}_1 + \dot{x}_2 = \dot{\hat{x}}_1 + \dot{\hat{x}}_2 = \dot{\hat{x}}_1$. However,

$$\dot{x}_1 + \dot{x}_2 = 2\dot{x}_1 = 2\,\frac{1}{4}\left(1 - \frac{\frac{1}{4}}{\left\lceil 1 - \frac{1}{4} + \frac{1}{4} \right\rceil}\right) = \frac{3}{8}$$

$$\neq \dot{\hat{x}}_1 = \frac{1}{2}\left(1 - \frac{\frac{1}{2}}{\left\lceil 1 + 0 + \frac{1}{4} \right\rceil}\right) = \frac{1}{2}\left(1 - \frac{4}{10}\right) = \frac{3}{10}.$$

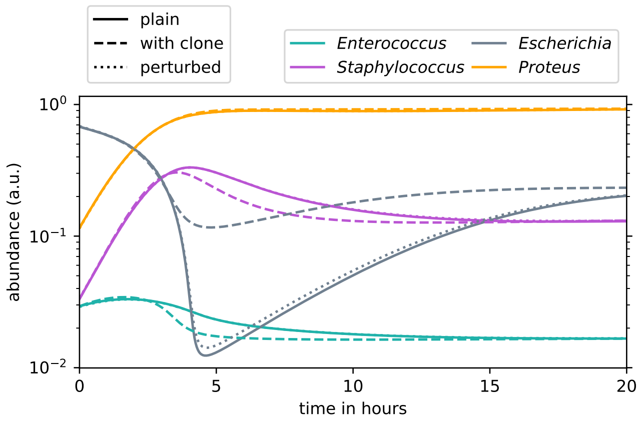

FIG. S1. Simulation of a UTI community (Community 3 from Ref. 1) using Eq. 21. Solid lines: plain simulation; each strain is represented by one population. Dashed lines: the same, except that there are two *Enterococcus* populations with identical parameters and half the initial abundance each. The abundance shown for *Enterococcus* is summed over these two populations. Dotted lines: like plain simulation, except that the initial abundances were perturbed by 1 % in a random direction. (A dotted line may be mostly covered by the respective solid line.) This demonstrates that the difference between the former two simulations is not caused by numerical noise and sensitivity to initial conditions. See Section B for details of the simulation and *S1 Code* for the simulation code.

B. Comparing the old and new UTI model with experimental data

To test the applicability of our UTI model (Eq. 26) in practice, we compared its predictions of in-vitro experiments with known outcome to those of the previous model (Eq. 21).

In the in-vitro experiments from Ref. 1 (cf. Fig. S7 B therein), each strain was inoculated at a fixed optical density ($OD_{600} = 0.001$) grown for $4 \times 30\,\text{h}$ and diluted by a factor of $\frac{1}{36}$ in between. Each experiment was performed in triplicate. Final abundances were determined from colony-forming units on Chromagar.

We mimicked this experimental procedure in simulations using both models. For these, we use the data from Ref. 1 as is, with the exception of data describing interactions between two identical strains: First, we assume that a strain cannot grow on the portion of the medium that is its own supernatant, but only on the portion that is fresh medium, which makes up $1 - v = 0.6$ of the medium. We set $c_{ii} = 1 - v$ to adhere to this ideal. Second, in the medium conditioned by itself, a strain's growth rate should at most be the same as in an unconditioned medium and at worst be proportional to the concentration of nutrients, and thus to $1 - v$. We therefore

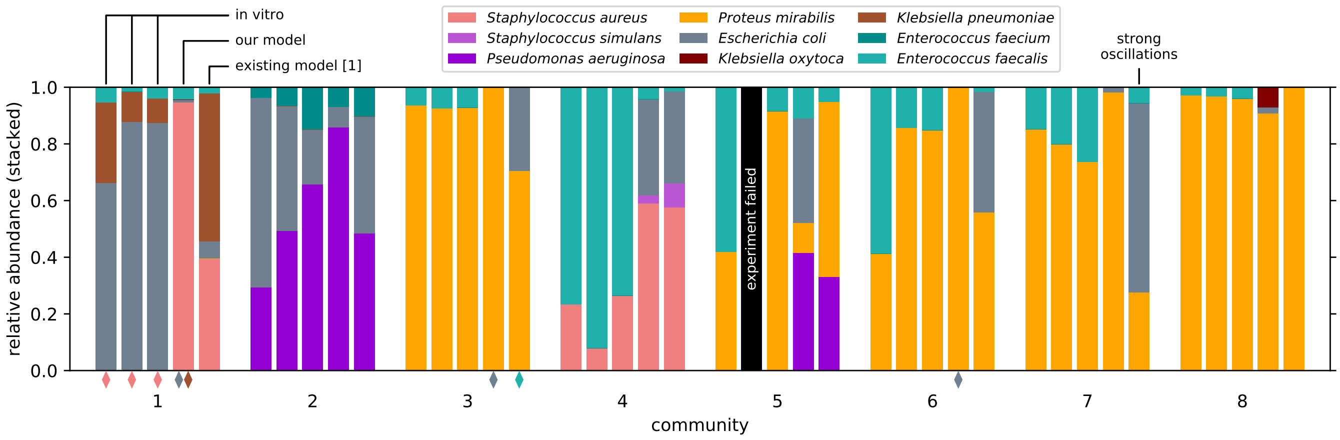

FIG. S2. Comparison of the final relative abundances of in-vitro experiments from Ref. 1 (Fig. S7 B) and simulations with our model (Eq. 26) and the model from Ref. 1 (Eq. 21). Communities are numbered as in Ref. 1. Diamonds at the bottom indicate an abundance of the respective population between 10^{-4} and 10^{-2}. Community 2 featured two strains of *Enterococcus faecium*, for which we only report the summed populations since they could not be distinguished in experiment. All simulations converged in the sense that they exhibited little difference between dilution steps – except for the model from Ref. 1 and Community 7, which exhibited strong oscillations.

restrict g_{ii} to the interval $[(1-v)g_i, g_i]$, i.e., we substitute g_{ii} with $\max((1-v)g_i, \min(g_{ii}, g_i))$. Without these adjustments to c_{ii} and g_{ii}, we would obtain implausible results, e.g., in case of $c_{ii} > 1-v$, the respective strain could never stop growing since it effectively increases the size of its own niche.

We performed all simulations with JiTCODE [2] using the LSODA method. See *S1 Code* for the simulation code. To obtain continuity as required by the integration method, we approximate $\lceil z \rceil = \max(0, z) \approx \frac{1}{2}\left(z + \sqrt{z^2 + 10^{-6}}\right)$.

We converted abundances in the simulation results to optical densities by undoing the respective normalization of abundances (fixing $c_j = 1$, see Fig. 4). We then converted the optical densities to displayed abundances (in Figs. S1 and S2) by approximating that optical density is proportional to biovolume.

The results of the simulation are shown in Fig. S2. Note that quantitatively predicting the population dynamics in experimental scenarios like this without in-depth knowledge about the involved microbes is a highly difficult challenge. Moreover, the high-throughput interaction data used to build the models is restricted; for example, it does not feature higher-order interactions, and the supernatant used to determine interactions will not contain toxins whose production is triggered by products of their target. Therefore, neither model can be expected to make perfect predictions. Due to this and given the low number of samples, we only visually evaluate similarity and refrain from a more detailed quantification. Also note that the goal is not to directly compare the results of the two models to each other.

We find that both models are in equally good or bad agreement with the experiment for six communities (1, 2, 4, 5, 6, and 8), while the predictions of our model (Eq. 26) are better for two communities (3 and 7). These results indicate that models satisfying our consistency criteria are at least equally suitable for describing community dynamics. Our results do not challenge the conclusions of Ref. 1, as both models yield similar results with respect to the key findings, in particular the ecological stability of communities, i.e., whether all populations in a community surivive (cf. Fig. S7 B in Ref. 1). This is plausible since both models have the same fixed points if the growth term is ignored and $c_{jk} < 1$ for all j, k and can thus be expected to yield similar final states. Note that in the serial-dilution scenario investigated here, differences between the models may also arise when the system is diluted before it has equilibrated.

References

[1] M. G. J. de Vos, M. Zagorski, A. McNally, and T. Bollenbach, Proc. Natl. Acad. Sci. U.S.A. **114**, 10666 (2017).

[2] G. Ansmann, Chaos **28**, 043116 (2018).
