# Supplementary material for: Building clone-consistent ecosystem models
Source: PLoS Comput Biol. 2021 Feb 8;17(2):e1008635. doi: 10.1371/journal.pcbi.1008635 (PMC7895417; doi:10.1371/journal.pcbi.1008635)
Supplement: Attachment — Submitted filename: Response.pdf [file pcbi.1008635.s004.pdf]

# Response to Reviewer 1

There are a few boxes left unchecked in the reproducibility report, for which we are not sure whether they reflect a problem at all, what exactly the problem is, and what actions they imply. We here briefly respond to them:

> *The initial conditions are provided for each of the simulations*

The initial conditions of Figs. 1 and 5 (now S1) are arbitrary and purely chosen for illustrative purposes. They are named variables in the source code, and in the manuscript, they can be easily read from the plots. For Fig. 6 (now S2), deriving the initial conditions is more complicated, as they have to mimic the experimental scenario. Achieving this requires a translation between normalized abundance and OD. However, all required information is in Supplement S2 and the code reflects this with named variables and methods.

> *Details for creating reported graphical results from the simulation results*

The provided scripts directly produce the plots used in the paper, so all the technical details are there. We consider the visualisation sufficiently straightforward that it requires no specific elaboration in the manuscript.

> *Machine-readable tables of parameter values*

These are all the MAT files (and we now briefly describe them in a readme file).

> *All simulation experiments are fully defined (events listed, collection times and measurements specified, algorithms provided, simulator specified, etc.)*

We specified the simulation software and algorithms and integrator in Supplement S2. We presume that *collection times* refers to sampling times. These times are appropriately dense for Figs. 1 and S1 (which is not worth mentioning); they are not relevant for Fig. S2 as only the final state is shown.

> *Conform to standards: Insufficient*

This is vague, even when considering the rubric, which says: "Adopt and promote generally applicable and discipline specific operating procedures, guidelines, and regulations accepted as best practices." We are confident that we conform to the standards of the field and would need more specific criticism if anything needs to be changed.

# Response to Reviewer 2

> *A basic requirement of any mathematical model is that it should provide consistent results if applied to the same situation. While this would have been of intuitive to the founders of theroretical ecology, it seems to be increasingly forgotten in more recent models that can accomodate a flexible number of species. In those models one could arbitrarily split a species into two model species and would expect the same result as if they were represented by one model species. But not all current models meet this essential requirement. The authors present a novel framework by which this requirement can be met in practice.*
>
> *Many readers will feel that they know and use these concepts already, but a large enough fraction of modellers seem to be unaware of it tha this publication is valuable.*
>
> *The manuscript leaves very little to criticise in terms of content or presentation and would be publishable in the present form.*

We thank the reviewer for his positive and encouraging assessment.

> *However, let me mention a subtle point: The author's want to make sure they avoid the impression of being naive with regards to the intentions of model designers. Suppose I want to model a number of stably coexisting populations that compete for a common resource. I might write*
>
> $$dR/dt = R_{in} - \sum(a_i R X_i)$$
> $$dX_i/dt = a_i X_i R - X_i^2$$
>
> *Of course this model violates the clone consistency conditions. The authors would argue that I have made a mistake and should have used loss terms of the form* $X_i(\sum X_j)$ *instead of* $X_i^2$. *This is in principle a valid point and many readers will profit from seeing it. However, for the sake of argument I want to defend the* $X_i^2$ *terms. I stated above that I want to model stably coexisting populations. We know that asymptotically stable coexistence is only possible if the different species occupy distinct niches (modeled in this case by the square terms). In this case the clone consistenty argument formally does not apply. I cannot argue that I can arbitralily split a population in two and represent it by two model variables, because the resulting populations would occupy the same niche and are hence outside the scope of my model.*

*As another example: If this were taken to far one could criticise Bob May's famous 1972 random matrix model for not being clone consistent. The model describes species interactions by a random matrix where elements are drawn independently. But if I would split a species in two arbitrarily the corresponding rows and columns would be correlated.*

*My point is that if I intend to model populations in different niches, this intention alone already removes the need for clone consistency in the model. (Conversely of of course one should be aware that violating clone consistency as doing so inserts arbitrary niches and thus constitutes an implicit assumption as the authors point out.).*

*The takeaway from is that one cannot decide whether a model is good or bad by looking at the equations alone, because whether clone consistency is even a criterion depends on the detailed premises of the model. The authors are right when they point out it would be bad to violate clone consistency accidentally without being aware of the additional assumptions that this implies. But, on the other hand, I would be equally bad to enforce clone consistency when it is at odds with the explicitely states assumptions. Doing so would prevent us from exploring some very valuable mathematical models.*

*The wording in some places suggests that the authors are aware of this point, but I fear that it would be lost on the casual or less informed reader will probably miss this. It would be good to mention it prominenently (e.g. when the authors present their introductory example) and perhaps hint in the abstract and/or author summary that sometimes clone inconsistecy is intentional and very much desired.*

We agree with the reviewer that it is important prevent an overzealous application of our rules. We would like to emphasize that we already devote an entire subsection of *Implications* (ll. 362 ff) to discuss the limitations of our approach and how it can point to assumptions such as the distinct niches. Indeed, the reviewer's first example is equivalent to the one discussed in Eq. 27 (formerly Eq. 26). As for the second example (May's random matrix model), we concur with the reviewer that applying our work in the suggested way would be profoundly wrong and pose a severe misinterpretation of our work. However, we think that addressing such an example in the manuscript would require extensive elaboration and could easily become more confusing than helpful, in particular since the model does conform with our criteria and the presumed clone inconsistency arises in a different way.

Following this reviewer suggestion, we now reference our discussion of this issue more clearly early in the manuscript, specifically we now

write in the abstract: "Although clone consistency can always be achieved with sufficient assumptions, we argue that it is important to explicitly name and consider the assumptions made: They may not be justified or limit the applicability of models and the generality of the results obtained with them." And in the author summary: "We further discuss that clone inconsistency, which occurs in several prominent models, reflects strong, often implicit, assumptions and it is important to check whether these are justified."

## Response to Reviewer 3

> *I consider the constructive framework developed by the authors and its translation into instructions on how to build consistent models, a valuable contribution to the field of ecological population models. However, I have some major concerns regarding the presentation of the work, its lack of context with respect to previous results in this area and the clarity and completeness of the interpretation of the results in the context of nonlinear dynamical systems. These major issues would need to be addressed for the article to proceed. They are detailed in the following together with several minor points. Numbers in parenthesis refer to the line number in the manuscript.*

We thank the reviewer for their assessment and the detailed constructive criticism. We have addressed all concerns, as described in detail below.

### *Issue 1:*

> *While the constructive framework appears to be plausible, the soundness of its justification suffers from unclear language, making it difficult to follow. I advise the authors to get feedback from colleagues with little familiarity with the work or even to work with a writing coach to improve the flow and readability of the text. The language should also be revised in terms of scientific writing standards. For example, I do not believe 'inevitable' to be the proper vocabulary to describe properties of mathematical objects. Furthermore, the authors should decide on a target audience and reflect this in their presentation. The presentation is uneven in terms of the required background knowledge in order to comprehend the statements.*

We agree that this work needs to be easily accessible. The manuscript was already reviewed by several colleagues and others. Following the

reviewer's suggestion, we obtained another round of feedback from an uninitiated colleague and made numerous changes to make the text easier to follow.

It is intentional that *Methods* is more mathematically challenging than the main manuscript. Our intent is that everybody who may want to apply our results can understand the main manuscript (without *Methods*). In contrast, *Methods* contains the mathematical details required to arrive at these results, which require more mathematical expertise. At the end of the introduction (ll. 112 f), we now explicitly state that *Methods* is intended for the mathematically inclined reader.

We removed two instances of the word *inevitable* (ll. 86 and 432) since they did not add much. However, we kept it in: "Thus, the model violates our consistency criteria and the observed clone inconsistency (Fig. 1) is inevitable." (ll. 228 f). Here, *inevitable* does not describe a mathematical property, but concisely stresses the strong connection between a modelling "mistake" and its consequences: A model not complying with our criteria will certainly exhibit inconsistencies like in Fig. 1. We could replace *inevitable* with synonyms like *inescapable* or *unavoidable,* but we do not consider these any better.

### *Issue 2:*

> *While the authors provide accessible guidelines on how to construct a consistent model and how to check existent models for consistency, the manuscript fails to state and clarify the mathematical properties that allow models of the proposed form to be consistent. In the authors summary the authors claim to have 'investigated the mathematical properties of clone-consistent models', but I do not see where in the manuscript these mathematical properties are described. The authors do provide criteria that impact functions should fulfil, but never explain why they do so or actually show that they do.*

As we understand the reviewer's concerns, we devoted entire sections to them, which the reviewer is clearly aware of, going by their accurate summary of our results. Here is our direct answer to the reviewer's concerns as we understand them:

The mathematical properties of clone-consistent models are:

- Impact functions (i.e., elementary ingredients of clone-consistent models) form a functional algebra ($\Phi$) generated by linear combinations and constant functions. If and only if a function is in that algebra it fulfils our criteria.

- Models of population growth additionally must be of the form described by Eqs. 10 and 11.

Consequences and more accessible formulations of these properties are described throughout the section: *What models are clone-consistent?* Being built from linear combinations and constant functions (via addition, multiplication, and function application or taking a limit, respectively) is what allows impact functions (or the models using them) to be consistent.

We made changes throughout the section *The functional algebra of impact functions* to make these connections clearer.

> *In addition, how the properties of the impact functions translate to the properties of the whole model and especially the solutions of the associated differential equations is left unclear. Furthermore, the authors state not to be able to make statements about the cause of inconsistency (line 392). I am wondering how the cause of inconsistency is connected to the fact that a nonlinear differential equations is unlikely to show additivity in the systems state variables (in the sense that* $f(x) + f(y)$ *is not equal to* $f(x + y)$*) unless it is built in a way that is has this property? Just as the actual cause of the inconsistency in the motivating example on lions is not that logarithms are not allowed in impact functions but that these system equations are not additive in* $x_2$ *and* $x_3$*. In summary, this comes down to a lack of the discussion of the framework in the context of nonlinear dynamical systems, which is also closely connected to the next issue.*

We do not expect that clone consistency or the use of impact functions allows for drawing general conclusions about the respective models, including about their dynamics. For example, conclusions such as "Clone-consistent models are less likely to exhibit oscillations" are unlikely to follow from properties of impact functions alone. Even if such conclusions were possible, they would not be straightforward to draw and go beyond the goals of our work. We now elaborate on this point in more detail in the first paragraph of *General implications* (ll. 430 ff). In particular, we explicitly discuss that clone-consistent models can still be non-linear: "Not only can a non-linear function still be applied to the linear combination (e.g., $\chi$ in Eq. 6), but any existing non-linear model can be made clone-consistent by sufficiently strong assumptions. The central question is whether these assumptions are biologically justified."

### *Issue 3:*

> *The manuscript fails to address how the findings relate to previous research in the area of nonlinear differential equation model reduction based on the aggregation of several state variables into a single one. If the goal is to preserve the system dynamics exactly this is for example termed 'perfect aggregation' in the field of ecological modelling, or 'exact lumping' in the field of biochemical reaction network modelling (just to mention two out of the many fields). The aggregation of variables via sums, as also considered by the authors, is a special case of what is called aggregation by a linear map or linear lumping in the context of the aforementioned fields, respectively. The following references provide an overview on this topic but are by no means exhaustive:*
> *Tóth, J., Li, G., Rabitz, H., & Tomlin, A. S. (1997). The effect of lumping and expanding on kinetic differential equations. SIAM Journal on Applied Mathematics, 57(6), 1531–1556. [URL]*
> *and references therein, especially*
> *Iwasa, Y., Andreasen, V., & Levin, S. (1987). Aggregation in model ecosystems. I. Perfect aggregation. Ecological Modelling, 37(3–4), 287–302. [URL]*
> *and references therein.*
>
> *I am aware that the framework proposed by the authors goes beyond the aggregation of identical species to also include consistency regarding the aggregation of identical modes of interaction only, but I still believe that the manuscript would profit from a discussion of the connection to the above-mentioned work.*

We thank the reviewer for pointing out this prior work. These works consider the conditions under which models can be simplified when some dynamical variables are redundant (or near-redundant). A typical case for this is indeed that a model is clone-consistent and there are actual clones in the model. In contrast, our work considers the properties a model must have for this situation to arise in the first place. Therefore, these works and ours are related but not special cases of each other. An illustrative difference is that parameters that describe how populations affect each other play a crucial role in our work but not in the references mentioned.

Nevertheless, we agree that it is helpful to mention this previous work and explain the differences to our approach. In the revised manuscript, we thus describe the relation between our and the suggested works in the introduction, writing: "Finally, if a model is clone-consistent and clones

actually exist, it can be simplified; this is called *aggregating* or *lumping* [citation of Tóth et al. and Iwasa et al.]."

## *Examples*

***Lack of reader guidance:***

*In general, the manuscript in several places lacks paragraphs to guide the reader. With that, I refer to paragraphs especially in the beginning of a section that prepare the reader for what results to expect in this section and which plot out the strategy on how the authors will get there. A general 'the paper is structured as follows ...' paragraph mentioning what to find in which section would also be helpful. For example, the section 'What models are clone consistent' right away starts out with impact functions without any information on how these functions relate to the models that the authors set out to find. Other examples are 'The Functional Algebra of Impact Functions' (490) and 'Deriving a New Model for UTI strains - the Legwork' (598).*

We agree that more guidance for the reader is helpful. Following the reviewer's suggestion, we rewrote the last paragraph of the introduction (ll. 103 ff) to guide the reader along the manuscript's structure and added short introductions to many sections (ll. 116 ff, 273 ff, 512 ff, 635 f, 652 f) including those mentioned by the reviewer.

***Problems in manuscript structure:***

*Figure 3 cannot be understood since needed information is only given later in the manuscript. Upper part of the figure: The reader cannot understand why question 5 is answered the way it is, because the checked model is only introduced and discussed later in the manuscript. This is especially a problem for question 5, since the answers to this question cannot be interpreted without the information given later in the manuscript. The answers to question 5 are confusing as well since it is not clear whether the answer is yes (no) or depending on the application.*

We thank the reviewer for pointing this out. The purpose of Figure 3 is to explain the general procedure for validating clone consistency. To this end, we show a specific example with arbitrarily chosen answers to the binary questions (3, 5, and 6). We did not intend that the reader understands the specific example answers we used for Question 5 at this point and do not consider this necessary to understand the general scheme. We

now label the answers to Question 5 as "arbitrary example" to make this clearer.

> *Finally, when the question of ecological plausibility is dealt with in the manuscript, the connection to question 5 (or Figure 3) is not indicated (line 294 ff).*

Thanks for pointing this out. We now refer back to Figure 3 at this point (l. 325 f).

> *The authors also might want to reconsider the relevance of question 4, since later in the manuscript (line 340) it is stated that rewriting an equation in this way is always possible.*

Good point. We changed Points 4 and 5 of the recipe to: "If not, cast them into this form by choosing parameters to be zero." and "Are these choices biologically justified? (Depends on application.)"

> *Lower part of Figure 3: The meaning of the footnote within the figure is unclear.*

We tried to clarify this footnote, which now reads: "This part only applies to models of population growth as in Eq. 10".

> *In question 2 omega which was previously defined as a basic impact function is used as symbol for a function of a basic impact function.*

We fixed this and now use $\chi$ in analogy to Eqs. 6 and 13.

> ***Inconsistency between section title and section content:***
>
> *The section 'Implications contains' contemplation on various aspects of which most content wise fit in neither of the subsections titled 'Checking Clone Consistency to Reveal Implicit Assumptions' and 'General Implications for Model Design'.*

We restructured this section and improved the subsection titles.

***Ambiguities making it hard for the reader to grasp the essential concept:***

*The models described by equations (2) and (3) cannot be the 'same model' (line 68 and caption of Figure 1) since one of them has two state variables while the other on has three.*

We now use *general model* instead of *model* to make clear that this refers to Eq. 1 (in which the number of populations is a parameter) and not a specific realisation of this general model (as described by Eqs. 2 and 3). We refrained from removing this formulation altogether since the fact that the simulations are based on the same general model is crucial here and this wording clearly communicates this.

***Incomprehensive paragraphs:***

*The paragraph starting from line 217 on building models with aggregated phenomenological observables:*

We rewrote and restructured this entire paragraph, addressing all the criticisms of the reviewer, in detail:

- *223: 'each of the $m$ experimentally determined interaction parameter'. Not clear what is meant with interaction parameters here. Each single measurement? Interaction parameter seems to have a different meaning here than in line 42.*

We now clarify that $m$ is "the number of experimental interaction observables, i.e., the number of measurements per (ordered) pair of populations" (ll. 243 f).

- *What is the reasoning behind the general Ansatz in equation (13)?*

We now start off with an even more general ansatz, and the former Eq. 13 (now Eq. 14) becomes a specific case. We elaborate that this ansatz arises from "[c]ombining Eqs. 10, 11, 6, and 5" (l. 240).

*The section on how to determine parameters and functions (224ff) is unclear.*

We added examples to better explain this process (ll. 254 f) and also expect that the way we restructured the entire paragraph will help the reader to better connect this part to the context.

- *231: What are building blocks in this context?*

We replaced *building blocks* with *basic impact functions* (ll. 243 ff) to avoid this confusion. We also expect that the restructuring of the paragraph helps to clarify this.

- *234: 'Finally, for some applications, a sum or more complex way to combine the basic impact functions may be appropriate (as opposed to the product used in Eq. 13). In New Model, we provide an example for this approach.' What approach? The product version or the sum?*

This refers to the entire approach outlined in the paragraph (though a product is used in this particular case). We now explicitly refer to the "approach" (l. 260). Further, the restructuring of the paragraph avoids that this reference is mistaken.

> ***Non-causal relations between sentences that imply such relations:***
>
> *Line 47: 'These new experimental scenarios often call for new ecological models that can incorporate the respective data. One reason for this is that there is no single answer as to how multi-parameter o higher-order interactions should be measured [3, 6, 16, 20, 23].'*

We now clarified this sentence, writing in lines 49 ff: "These new experimental scenarios call for new ecological models that can incorporate the respective data. Existing models are often not suitable here since there is no uniform answer as to how multi-parameter or higher-order interactions should be measured [citations]."

> *Overall meaningless sentences like: 'Moreover, clone-inconsistent models are diverse for the same reason that non-linear functions are.' (394)*

We expanded the respective paragraph and instead of this sentence, we now write: "For illustration, the diversity of clone-inconsistent models may be compared to that of all numbers not divisible by seven."

We do not consider this sentence meaningless, as it can help readers to appreciate the diversity of clone-inconsistent models and why it is difficult (if not impossible) to make general statements about them.

## *Minor issues*

### *Main Text*

1. *It is not clear to me why the authors come up with a new label (clone consistency) for something that has been described before. The authors state mere similarity to earlier descriptions of the problem (line 59). If there is indeed a difference between earlier descriptions of the problem and 'clone consistency', I would like to request a discussion of those differences by the authors to justify new wording.*

From the existing labels we know (all cited in the introduction), we consider *invariance under relabeling* to be the best candidate. However, this label is not established in the field (it is only used by Murrel et al (2004) as far as we know) and we considered it too wordy and grammatically inflexible for the heavy usage required in our manuscript. For example, instead of *clone inconsistency* we would have to write something like *lack of invariance under relabeling* or *lack of relabeling invariance.*

Regarding the other alternatives:

- Kuang (2002) and Arditi and Michalski (1996) use *invariant under identification of identical species,* which we consider too cumbersome. This is corroborated by both papers referring to it as *Criterion 1* instead.

- Drossel et al (2001) uses *invariance under aggregation of identical species.* Like the above, we consider this too cumbersome.

- Rossberg (2013), van Leeuwen et al. (2013), and Vallina (2014) use *"common-sense" criterion,* which is cumbersome, not descriptive, and bears the risk of coming off as arrogant in the context of our manuscript.

- Weizman's (1992) *twin property* is specific to diversity measures.

Hence, we prefer to keep the label *clone consistency.*

2. *It is not clear if the proposed framework can also be used for models that model the mechanism of interaction, e.g. for consumer resource models. If so, how does the proposed framework integrate with commonly used impact functions?*

While most of our examples focus on models where all the dynamical variables are populations, we never restrict ourselves to this case. In *Implications,* we discuss the application of our framework to consumer–resource models and similar (ll. 412 ff) and how impact functions relate to mechanisms (ll. 384 ff, 424 ff, and 445 ff.). The extension of our framework in this direction is straightforward: one simply uses impact functions to describe the impact of an ecosystem on a resource instead of a population. We now included references to consumer–resource models and similar at appropriate points in the manuscript (ll. 128 f and 210). Also see the next point.

> *The authors should unambiguously state in the introduction the latest for which types of models this framework can be used, so readers can decide early on whether the paper is relevant for their research.*

To clarify this point, we now write in the introduction (ll. 112 f): "we discuss how our framework applies to all models involving multiple populations". We understand that this is rather vague. However, the more precise answer "every model that contains impact functions" is not understandable to the reader at this point. Also, while there are models to which our framework applies in general, but for which there is no danger of clone inconsistency (e.g., mechanistic models featuring interaction mechanisms that are exclusive to a pair of species/resources), we again cannot reasonably discern these cases without explaining central parts of our framework.

> 3. *115, 123, 203,… the Volterra model [1]): not clear to which model/ equation in this reference the authors refer to. What is the difference between what the authors call the Volterra model and what is commonly referred to as the generalized Lotka-Volterra model?*

They are the same. We feel that *generalized Lotka–Volterra model* is a bit of misnomer, since Volterra already considered this model (Ref. 1, p. 42) and there was no need to generalize it. However, we concur that it will be clearer for most readers to use the established term, so we changed it to *generalized Lotka-Volterra model* (ll. 132, 140, 222 and 362 f).

> 4. *In the authors summary the authors state that something along the lines of that one way to test if a models make sense is to check if the dynamics stay the same if you split a model species. I do not agree that this is either a necessary or a sufficient condition to decide on the validity of a model.*

We agree with the reviewer and that was not the intended meaning. To avoid this misunderstanding, we now write: “One sanity check for such models is to virtually split a population […]” (ll. 21 f).

5. *The authors should clarify how their results for the system translate to properties of fixed points*

We presume that this refers to the case study. As we elaborate at the end of the Supplement S2, the fixed points of the two models are equal, if we assume that the growth term does not become zero and $c_{jk} < 1$, which is the predominant case. If $c_{jk} > 1$, things become complicated due to case distinctions in the existing model (Eq. 21) though a certain similarity of fixed points can be expected. We now briefly reference this in the main manuscript (ll. 358 f).

We do not expect a more detailed analysis of this point to be worthwhile, in particular since it is not central to our manuscript. Also see our response on the general possibility of deducing dynamical properties from clone (in)consistency above.

### *Methods*

6. *As indicated to the comment on reader guidance, the methods part would also benefit from more supportive text to help the reader understand what to expect from the section.*

We now explicitly refer to the subsections of *Methods* in the main manuscript to point the reader directly to the right subsection and briefly describe the contents of the *Methods* section at the end of the introduction (ll. 113 f). With the exception of *The functional algebra of impact functions* and *Proof: $\Xi$ generates $\Phi$* (which are clearly linked), the subsections of *Methods* have no overarching narrative. Thus, an introductory paragraph would mostly contain subsection titles. Instead, we reworked the first paragraph of each subsection in *Methods* to better guide the reader.

*The subsection about notation indicates a comprehensive symbolic language that is used rigorously. This is not the case e.g.:*

1. *the use of mathfrak symbol sequences in Definition 1.*

We now clarify that the notation overview does not fully extend to *Proof: $\Xi$ generates $\Phi$* (ll. 509 f), as it requires a considerable amount of special notation that is only relevant to that subsection and would only confuse readers who do not engage with it.

2. *the quantifiers are used inconsistently, i.e. after an expression in (30), before an expression in (38)*

We moved all quantifiers to the front or replaced them with prose.

3. *The appearance of the triple bar symbol in (40)*

We presume that the reviewer sees this in conflict to our usage of ":="; we avoided this notation.

7. *The overall presentation indicates a formal approach by applying a Definition-Theorem-Proof style. This is not consistently applied, e.g.:*

1. *the domains and codomains of the impact functions or the functions $\mathcal{R}$ are never given*

The definition–theorem–proof style is exclusive to the section *Proof: $\Xi$ generates $\Phi$* and we do not consider it useful to extend it to the section in question *(Non-impact-function contribution to abundance changes must be proportional).* However, we reworked that section and now clarify the domains and codomains of $\mathcal{R}$ (l. 637).

2. *the informal introduction of the terminology "point-separate" which appears to be the same as "separate" in functional analysis textbooks*

We replaced *point-separate* with *separate points.* While a plain *separate* is used as well in the literature, we consider a more distinct term more expedient here.

8. *The section 'Non-Impact-Function Contribution to Abundance Changes Must be Proportional' seems to reproduce the textbook argument that additivity plus continuity implies homogeneity, see e.g. Linearity on Wikipedia. It leaves out the decisive steps from that argument. Because of both, the involved way of going to the linearity in the first argument and the obscure presentation, the value of the paragraph for the reader is unclear. Without already knowing what the section is about, the section title is hard to interpret.*

We considerably reworked this section, improving its connection to the main text, the notation, and elaborations of our line of thought. However, we do not intend to reproduce "additivity $\Rightarrow$ homogeneity" here. We consider it sufficient to reference this well-known result (which we now do in lines 646 ff).

9. *Paragraph from 505- 509. The authors need to clarify how this is connected to what the reader has learned about impact functions up to this point. What is meant by 'capture the application'?*

We rephrased the respective sentence and now write in lines 551 ff: "In application, the fact that $\Phi$ is a closed functional algebra, i.e., that limits remain within it, is relevant as it addresses the case of a (non-polynomial, continuous) function being applied to the result of an entire impact function or the parameters."

### *S2 Comparing the Old and New UTI Model with Experimental Data*

10. *The statement "a strain cannot grow on the portion of the medium that is its own supernatant" (line 677) is not generally true. Without the respective experiments, this statement cannot be made (a fact that the authors seem to acknowledge in line 255). If a species has grown to saturation in a medium this does not necessarily mean that growth saturated due to complete exhaustion of an essential nutrient. Growding effects for example also play a role. If the authors need to use this as an assumption in their model they need to formulate is as such.*

We clarified that this is an assumption (S2, ll 47 ff).

11. *680: 'Second, in the medium conditioned by itself, a strain's growth rate should at best slightly lower than in an unconditioned medium ...'*
    *This is not a generally valid statement. It depends very much on whether nutrients are in excess in your medium or not. As long as the nutrients in the mix of conditioned and new medium are not limiting the maximum relative growth rate will also be reached under these conditions.*

We removed the $1-\epsilon$ term here as it poses an unnecessary complication that has little effect on the results (S2, ll. 55 ff).

*The authors should clarify how the $g_{ii}$ were chosen within the mentioned interval (line 683) for the simulations.*

We now clarify that "we substitute $g_{ii}$ with $\max((1-v)g_i, \min(g_{ii}, g_i))$" (S2, ll. 55 f).

12. *683: 'Without these adjustments, we would obtain implausible results, e.g., in case of $c_{ii} > 1 - v$, the respective strain could never stop growing since it effectively increases the size of its own niche.'*
    *The authors should clarify this sentence. It is not clear to me what the authors mean here with adjustments.*

We now clarify that this refers to the adjustments of $c_{ii}$ and $g_{ii}$ (S2, ll. 56 ff).

13. *The findings regarding better or worse agreement with the data in comparison to the old model are not comprehensible. Neither the measure based on which these statements are made nor the features that were used to assess agreement are stated.*

The statement that this part of the supplement supports is: "We find that this model can explain observed species abundances and ecological stability in a small experimental dataset [...] at least as well as the previous model [...]" (ll. 357 ff). This is admittedly not very precise, but neither do the limitations of the experimental data as well as the low sample size allow for a more precise claim, nor is one required for the conclusions we draw from this. For this reason, quantifying this similarity would not provide any additional insight. Instead, we only evaluate the similarity of results visually (which we now explicitly state in line 80 ff of S2).

> *Claims about the stability of fixed points are made without a suitable analysis.*

We clarified the respective statement, which should not be about dynamical stability, but about ecological stability, i.e., whether all populations of a community survive.

> *Causes for observed differences and agreements in model outcomes are put forward without justification through simulations. There is no way to compare the population dynamics of the two models since only simulation endpoints are shown.*

The goal of the simulations is not to directly compare the two models to each other (which we now explicitly state in line 82 of S2), but rather to compare them to the experimental results, respectively. This is currently the only way to evaluate the performance of the models. As the experimental results only feature final abundances, the temporal evolutions of the simulations have little bearing. (This would be different if most of

the simulations exhibited strong oscillations instead of converging on a fixed point, as then the timing of the dilutions could considerably change the result. That is also is why we mention that all but one simulation converge.) In this light, we also changed the only sentence comparing the models to: "Note that in the serial-dilution scenario investigated here, differences between the models may also arise when the system is diluted before it has equilibrated." (S2, ll. 95 ff)

14. *Caption of Figure 6: 'All simulations converged, except for the model from Ref. 16 and Community 7, which exhibited strong chaotic fluctuations.'*
    *The authors should clarify what they mean with converged and with chaotic fluctuations/ oscillations.*

We now clarify this and write: "All simulations converged in the sense that they exhibited little difference between dilution steps – except for the model from [de Vos et al. (2017)] and Community 7, which exhibited strong oscillations." We do not mention chaos anymore, since it has no bearing on the manuscript that these oscillations are chaotic (the inverse of the maximum Lyapunov exponent is larger than the total simulation time).

### *S3 files*

15. *The authors should provide a README file together with the data and code files. This file should state the content of each file (e.g. which is the file that contains the data that were declared to be available in these files and which file was used to create which figure).*

We followed the reviewer's suggestion and added such a read-me file.

### *Misspellings and miswording*

*The term 'point-separates' appears to be a literal translation of the German wording, please check whether this is consistent with the contemporary term in English.*

See above. Note that we are not even aware of any German term for this.

*'Shape' of equations (functions, models, … ) (144, 147, 151…). The shape of an equation refers to the graphical representation of an equation. In the context that shape is used here, form would be the correct word (c.f. standard form of an equation, factored form of an equation, …)*

Fixed.

> *An abstract object like a mathematical model cannot have a biological shortcoming (390) since it does not have a biology.*

We removed the word *biological.*

> *'Capture the application' (line 504) seems to be a literal translation of the German wording, unclear what is meant here.*

See above.

> *Line 73: predators -> predator*

We changed the respective phrase to: "Population 2 contains the predators." (l. 77)

> *Line 260: this data -> these data (data are plural)*

We changed this to *dataset* which we consider to be more clear anyway.

> *Line 387: target[t]ing -> targeting*
> *Line 417: general modelling -> generalized modelling (according to the provided reference)*
> *Equation 35: limits of the sum missing in last equation?*
> *512: There is no j in the equation that needs to be defined, but I think the definition for p is missing*
> *520: unitial -> unital*
> *524: unitiality -> unitality*
> *650: represent -> represents*
> *681: best slightly -> best be slightly*

All fixed.
