# Supplementary material for: Building clone-consistent ecosystem models
Source: PLoS Comput Biol. 2021 Feb 8;17(2):e1008635. doi: 10.1371/journal.pcbi.1008635 (PMC7895417; doi:10.1371/journal.pcbi.1008635)
Supplement: Attachment — Submitted filename: Response.pdf [file pcbi.1008635.s005.pdf]

Response to Reviewer 3

> *In the revised version of the manuscript, most of my issues have been addressed. However, in the following I try to clarify one of my original issues that I think has not been fully addressed. Furthermore, I list some points where I disagree and some that are still not fully clear to me.*

We thank the reviewer for their assessment. We have addressed all the remaining concerns, as described in detail below.

> *'We do not expect that clone consistency or the use of impact functions allows for drawing general conclusions about the respective models, including about their dynamics.'*
>
> *The conclusion that in a clone consistent model the dynamics must stay the same if populations are merged (or split) is a conclusion about the model dynamics.*

This issue seems to boil down to what exactly is meant by *dynamics*. We were referring to dynamics in the sense of dynamical phenomena as studied in the field of non-linear dynamics, such as oscillations or chaos – a system can exhibit different types of dynamics in this sense, but we have no reason to believe that clone consistency (or its violation) correlates with particular types of dynamics. We made the respective clarification in the manuscript more explicit (ll. 442 ff): "Similarly, it is likely not possible to draw general conclusions about the dynamic behavior of the clone-consistent models – such as favoring or suppressing oscillatory dynamics."

> *My point comes down to the problem that the manuscript discusses clone consistency in two different contexts:*
>
> 1. *Clone consistency as a property of impact functions, which is defined verbally (I4 main text) and formally in the paper (I4in Methods).*
> 2. *Clone consistent population models, i.e. clone consistency as a property of a population model (eqn. (10)). The criterion for clone consistency in population models is discussed verbally for example in the abstract and illustrated in the motivating example. Clone consistency of models is not defined formally.*
>
> *The manuscript draws connections between impact functions and population models, e.g. eqn. (11). How the clone consistency of impact*

functions determines the clone consistency of population models is not explained, because clone consistent population models are not defined. The authors should clarify this connection.

We now clarify that I4 is a special case of clone consistency, applied to impact functions (ll. 155 ff): "Note that this criterion is a special case of clone consistency as described in the introduction. Using impact functions complying with this criterion is therefore necessary for a model to be clone-consistent, but not sufficient (we will address clone consistency on the scope of the entire model later)."

Later in *Assessing and building models,* we clarify that combining clone consistency of the effects of a community (via impact functions) and the effects on a community (Eq. 11) ensures clone consistency of the entire population model (ll. 206 f): "[Our treatment of impact functions allows us to ensure clone consistency when modeling the effects **of** a community.] To ensure clone consistency of an entire population model, we additionally need to require clone consistency when modeling the effects **on** the growth of a population."

We are confident that this subtle point is now clearer. We do not think that a more formal definition of clone consistency at the level of a population model (than the one we give in the introduction) would be helpful, as it would be a purely technical exercise that does not offer relevant insights.

'We now use general model instead of model to make clear that this refers to Eq. 1 (in which the number of populations is a parameter) and not a specific realisation of this general model (as described by Eqs. 2 and 3). We refrained from removing this formulation altogether since the fact that the simulations are based on the same general model is crucial here and this wording clearly communicates this.'

I am not convinced that the addition of the word 'general' makes this clearer. What's the purpose of showing specific realisations if those do not relevant for the example? In my opinion, this could be easily made clearer by using only Eq. 3 (instead of Eqs. 2+3). Then, to distinguish between the two scenarios only the vector of initial conditions is needed in addition to equation 2.

We appreciate the suggestion to simplify this example. However, reducing the differences of the two simulations in Fig. 1 to differences of initial conditions would likely do more harm than good as it would obscure one of the key points of this example: A central implication of splitting a population is that the number of populations increases. If our implementation did not reflect this because we used a technical trick (setting some

population sizes to zero in the initial condition), this could easily lead to confusion and would certainly require additional explanation. Moreover, this trick requires the assumption that an absent population has no effect (I2). It is not helpful to unnecessarily burden the reader with this additional aspect at this point. Also note that, for this particular model, this assumption would even be wrong (since $\log(0) = -\infty$).

> *243: First we identify the number of basic impact functions m as the number of experimental interaction observables, i.e., the number of measurements per (ordered) pair of populations.*
>
> *This is sentence still has the potential to confuse a first time reader. The first part of the sentence is clear but in the second part 'number of measurements' sounds like it has something to do with replicates. I think it would be clearer to just give an example for an observables in addition to the first part of the sentence.*

We removed the second half of the sentence and now consistently use the term *interaction observables* for this concept. We now introduce this term in the introductory sentence of the paragraph (ll. 241 ff), where we also give an example.

> *Footnote in Figure 3: "This part only applies to models of population growth as in Eq. 10". I still do not get what this footnote tries to tell the reader. The figure panel is about building a model. So what is the alternative to a "model of population growth" here? When would the parts indicated by the footnote not apply?*

The alternative would be models that capture the impact of the community on something else, e.g., a model of resource consumption. We expanded the footnote to clarify this: "This part only applies to models of population growth as in Eq. 10 (as opposed to, e.g., models of resource consumption)."

> *In line 334 the authors state that TWO experimental interaction parameters are available, but equations 16 -19 are FOUR criteria that are referred to in 304 as 'one criterion per experimental interaction parameter'. Are there two or four interaction arameters? This seems still to be related to the ambiguous use of the term 'interaction parameter' in the manuscript. On the one hand used for modes of interaction (interaction observables?) and on the other hand referring to the actual parameter in an equation. The authors should also explicitly state in words for each criterion (Eq. 17-19) to which 'interaction parameter' it corresponds.*

To avoid this confusion, we made our terminology more consistent in this respect (see response above). Here, we now use *observables* instead of *interaction parameters*, which was indeed incorrect. We also labeled the equations with the respective observable they capture as suggested.

> *315 As the model clearly satisfies Criteria I1-I3, it therefore must violate I4... Where is the causality between these two parts of the sentence?*

The preceding sentence implies that the model violates one of our criteria (I1–I4). We now explicitly state this: "Thus the model must violate at least one of our criteria."

> *358: Moreover, the fixed points of both models are usually the same. What is meant by usually here?*

We now clarify that this is "under most conditions" and refer to the appendix for details.

> *543: Otherwise Ξ would generate more than impact functions. Not clear what 'more than impact functions' could be.*

We edited this sentence to clarify: "Otherwise Ξ would also generate functions that are not impact functions."

> *242: Here, we refrain from transforming the parameters in Eq. 14 Should this be Eq. 13? Otherwise, it would make sense to make this statement after Eq. 14.*

This refers to Eq. 13 and is an artifact of the restructuring. We removed the reference to the equation entirely since it is directly above the sentence in question, and *here* already makes this connection.
